# Biopolymeric Formulations for Biocatalysis and Biomedical Applications

**DOI:** 10.1155/2014/418097

**Published:** 2014-06-17

**Authors:** Magdy M. M. Elnashar, Tarek Kahil

**Affiliations:** ^1^Center of Excellence, Encapsulation & Nanobiotechnology Group, Polymers Department, National Research Center, El-Behouth Street, Cairo 12311, Egypt; ^2^Biochemistry Department, Medical School, Taif University, Hawyia, Taif 5700, Saudi Arabia; ^3^Microbial Chemistry Department, National Research Center, El-Behouth Street, Cairo 12311, Egypt

## Abstract

Three gel disks formulations prepared using chitosan (Chito) or polyethylenimine (PEI) followed by glutaraldehyde were prepared for biocatalysis and biomedical applications. The carriers have been used to immobilize lactase covalently and it was evaluated in terms of enzyme loading capacity and enzyme kinetics (km and Vmax). The Km of the Chito formulation was almost half that of the PEI formulations, which is favored in industries. On the other hand, the gel disks were evaluated in terms of their swelling kinetics and the gels' morphology using SEM. The mechanism of the three gels' swelling was also studied and it was found to be non-Fickian, where the mechanism of transport depends on both the diffusion and polymer relaxation, which are controlling the overall rate of water uptake. The Chito formulation was 2–5 folds and PEI formulations were 33–62 folds in terms of the swelling rate constant and the diffusion rate, respectively. These results were highly supported by the SEM. This study will help scientists to design the right polymer network for enzymes immobilization as well as control the surface area and the swelling power of the polymers for different applications such as drug delivery systems and tissue engineering.

## 1. Introduction

Hydrogel is a network of polymer chains that are hydrophilic and is sometimes found as a colloidal gel in which water is the dispersion medium. Anionic and cationic hydrogels are networked structures of polymer chains cross-linked to each other and surrounded by an aqueous solution [1, 2]. The polymer chains contain acidic groups (anionic) which deprotonate at high pH leaving a negative charge on the polymer; for example, carrageenan gel as an anionic polymer contains –OH and –OSO_3_H groups; in an acidic medium, it deprotonates, where the –OH and –OSO_3_H groups become –O^−^ and –OSO_3_^−^, respectively. On the other hand, the basic groups (cationic) protonate at low pH leaving a positive charge; for example, chitosan and polyethylenimine as cationic polymers contain –NH_2_ groups; in acidic medium, they protonate to –NH_3_^+^. In the presence of an aqueous solution, the polymer chains absorb water and the association, dissociation, and binding of various ions to polymer chains cause the hydrogel to swell. The swelling and shrinking properties of hydrogels are currently being exploited in a number of applications including control of microfluidic flow [3], muscle-like actuators [4, 5], filtration/separation [6], and drug delivery systems [7, 8].

Common uses for hydrogels include also scaffolds in tissue engineering; hydrogel-coated wells have been used for cell culture, environmentally sensitive hydrogels which are also known as “smart gels” or “intelligent gels,” as sustained-release drug delivery systems, and as biosensors, used in disposable diapers where they absorb urine, in sanitary napkins, in contact lenses (silicone hydrogels, polyacrylamides), in EEG and ECG medical electrodes using hydrogels composed of cross-linked polymers (polyethylene oxide, polyAMPS, and polyvinylpyrrolidone), in rectal drug delivery and diagnosis, and in encapsulation of quantum dots [9].

However, swelling of polymer gels is one of the classical problems in both macromolecular science and technology [10–13]. Flory and Rehner developed an equilibrium swelling theory by considering the balance between the mixing of polymer chains with solvent and the elasticity of the polymer chains [10]. Ritger and Peppas presented mathematical models for drug diffusion from hydrogels [11]. Tanaka and Fillmore have studied the swelling kinetics of spherical gels [12] by using an equation of motion of the gel network [13]. The study of swelling kinetics has been further extended to different geometries: long solid gel cylinders and thin solid disks [14–16]. In these cases, a theoretical analysis of the kinetics of gel swelling and solvent motion is based on the solution of coupled equations of motion for a network and solvent [17].

To our knowledge, previous reports have not to any greater extent dealt with polyelectrolytes complex (PEC) based on alginate and *κ*-carrageenan to covalently immobilize enzymes with an exception of our recent works [1–3, 18–23]. Gels based on these biopolymers were treated with protonated polyethylenimine or chitosan to covalently cross-link penicillin G acylase, invertase, inulinase, phytase, or lactase via glutaraldehyde as a mediator.

Michaeli's constant, *K*_*m*_, by definition is the substrate concentration at which the reaction rate is half of the maximum velocity. In enzyme industries, *K*_*m*_ is highly favourable to be minimal as it means that the enzyme could reach its maximum velocity using low concentration of substrate. Accordingly, our aim was to study the effect of different PEC formulations and their correlations on the gels' swelling kinetics (*K*_*s*_ and *q*%) and solvent diffusion rate (*D*) for a better design of PEC having low *K*_*m*_ values.

In this work, we prepared three formulations in the form of gel disks based on polyelectrolytes interactions, one formulation using carrageenan with chitosan and two formulations using carrageenan with polyethylenimine as follows:

- carrageenan/chitosan/glutaraldehyde (Carr/Ch/Ga);
- carrageenan/polyethyleneimine, 8 h/glutaraldehyde (Carr/PE, 8 h/Ga);
- carrageenan/polyethyleneimine, 10 min/glutaraldehyde (Carr/PE, 10 min/Ga).

The concept for modification of carrageenan with chitosan or polyethylenimine was different than that used by other authors who used this combination for cross-linking of carrageenan surface via formation of PEC to harden the gels and to immobilize enzymes via entrapment [9, 24]. For example, Boadi and Neufeld, 2001 [9], used alginate and carrageenan to entrap tannase and then cross-linked the gel beads with chitosan followed by glutaraldehyde, whereas Shukla et al., 2004 [24], used *κ*-carrageenan gel to entrap horseradish peroxidase and cross-linked the gel beads using KCl and PEI.

In our case, we imparted three extra benefits to carrageenan by using partially protonated polycations chitosan or polyethylenimine. The first is creation of a new functionality on the carrageenan, amino groups (free NH_2_); the second is improvement of the carrageenan gel's thermal stability by forming a polyelectrolyte complex (PEC) between the carrageenan –OSO_3_^−^ and the chitosan or the polyethylenimine –NH_3_^+^ knowing that only polyamines can improve substantially the carrageenan gel's thermal stability [23]. Thirdly, the free amino groups (–NH_2_) were then further activated with glutaraldehyde to covalently immobilize lactase. The carriers have been evaluated in terms of their swelling kinetics, enzyme kinetics (*k*_*m*_ and *V*_max⁡_) of the immobilized enzymes, and the gels' morphology using SEM. A correlation between the swelling kinetics of the gel and its gel surfaces was compared with the enzyme kinetic constant's *K*_*m*_. The obtained results were useful for designing carriers that could be useful for immobilization of industrial enzymes, tissue engineering, and drug delivery systems.

## 2. Materials and Methods

### 2.1. Materials

*κ*-Carrageenan (Mw: 154,000; sulphate ester ~25%) and chitosan were supplied by Fluka. Lactase (EC 3.2.1.23) from* Aspergillus oryzae*, 11.8 U/mg, and polyethyleneimine were purchased from Sigma-Aldrich. Other chemicals were of Analar or equivalent quality. Parallel plate equipment was made in our laboratory for uniform gel sheets preparation. The gel disks dimensions were measured using a micrometer (Micro 2000, 0–25 mm). For mechanical strength (MS) measurements, INSTRON instrument (model 5564) was used in compression mode.

## 3. Experimental Techniques

As a general rule, all experiments were carried out in triplicate and data are means ± SD (*n* = 3).

### 3.1. Preparation of *κ*-Carrageenan Gel Disks

*κ*-Carrageenan gel was prepared as previously reported by Elnashar et al. 2008 [23] by dispersing 2% (w/v) carrageenan in distilled water at 70°C.

**Figure 1:**
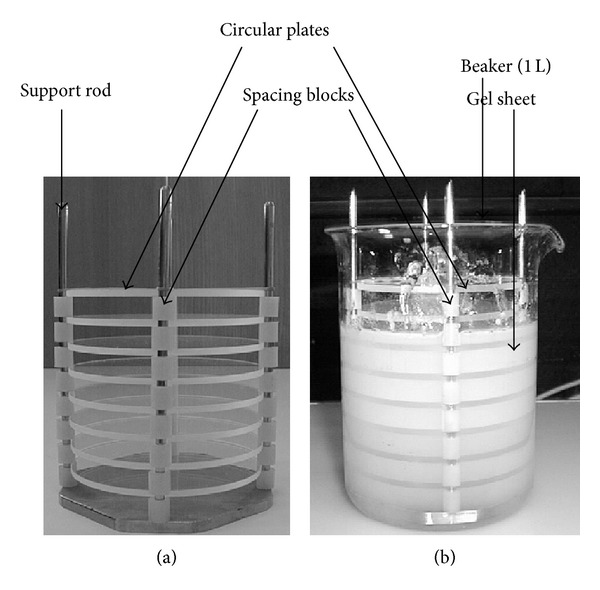
Parallel plates' equipment for making uniform gel disks.

The carrageenan solutions were mixed thoroughly using an overhead mechanical stirrer until complete dissolution had occurred. Glass parallel plates equipment designed by Elnashar et al., 2005 [25] as shown in Figure 1, with 10 mm gaps was then immersed into the hot molten gel to produce uniform gel sheets. Typically, 10 mm diameter gel disks of average weight 0.75 g were produced for immobilization.

The 10 mm thick gel sheets were cut into disks using cork borers for enzyme immobilization. Typically, 10 mm diameter gel disks, of an average weight of 0.75 g, were produced for immobilization.

### 3.2. Carrageenan Coated with Chitosan or Polyethylenimine and Schiff's Base Formation

In this experiment, the carrageenan gel was hardened with a 2% (w/v) chitosan (Ch) for 2 h or alternatively with 4% (v/v) polyethyleneimine (PE) for 10 min and 8 h. The modified gel disks were thoroughly washed with distilled water and soaked in 2% (v/v) glutaraldehyde (Ga) solution for 3 h. In this step, Ga acted as a Ch cross-linker, improving the gels' thermal stability [26] and as a spacer arm offering free aldehyde groups to react with the enzyme's amino groups via Schiff's base formation. The modified gel was thoroughly washed with distilled water to get rid of any unbound glutaraldehyde molecules. The FTIR of these gels was done in our previous articles, and they showed the formation of Schiff's base as well as the appearance of the free aldehyde groups required for covalent immobilization of the enzyme at the range of 1670 cm^−1^–1720 cm^−1^ [21–23, 27]. The reusability test revealed no leakage of the enzymes from the carriers after 15–20 reuses, which proved the immobilization using the covalent bond.

#### 3.2.1. Gel Disks Mechanical Strength Measurement

Gel disks of 10-mm height and 14-mm diameter were subjected to compression using an INSTRON model 5564 mechanical strength testing machine. The gel strength was measured as the critical compression force, the force required to rupture the gel disk. The test pieces were placed on the INSTRON bottom plate and compressed at a speed of 10 mm/min under a load of 1 N. As the disk was compressed (strained), it exerted an increasingly higher reaction force against the compression. Beyond a certain deformation (stress), the material can no longer resist and fractures. The mechanical strength of the sample was calculated from load per unit area of the disk (kg/cm^2^) at the fracture point. Samples were used in triplicate and data are means ± SD (*n* = 3).

### 3.3. Immobilization of Lactase and Soluble Protein Determination

Lactase was immobilized onto the modified gel disks. Two disks of each carrageenan (Carr) formulation

(d) carrageenan/chitosan/glutaraldehyde (Carr/Ch/Ga);
(e) carrageenan/polyethyleneimine, 8 h/glutaraldehyde (Carr/PE, 8 h/Ga);
(f) carrageenan/polyethyleneimine, 10 min/glutaraldehyde (Carr/PE, 10 min/Ga)

were washed thoroughly with distilled water and were incubated into 5 mL of enzyme solution (10 U/mL) prepared in 100 mM citrate-phosphate buffer at pH 4.5 for 16 h. The immobilized enzyme was washed thoroughly with the buffer solution containing Tris-HCl to block any free aldehyde group and to remove any unbound enzyme. The immobilized enzyme was stored at 4°C for further measurements. The supernatant and the wash were kept for soluble protein assay via bovine serum albumin (BSA) as a standard protein. The amount of immobilized enzymes was calculated per gram gel disks (U/g) according to the following equation:

(1) $$\begin{array}{c} E.L.C.=\frac{\left(M_{o}-M_{f}\right)}{W}, \end{array}$$

where *M*_*o*_ is the initial enzyme activity (U), *M*_*f*_ is the enzyme activity of the filtrate (U) after immobilization, and *W* is the weight of gel disks (g).

### 3.4. Lactase Activity Assay

Activity of lactase was determined by the rate of glucose formation in the reaction medium. Known amounts of immobilized or free enzyme were incubated into 10 mL of 200 mM lactose solution in citrate-phosphate buffer (100 mM, pH 4.5) for 1 h at 70°C and 100 rpm. One enzyme unit (IU) was defined as the amount of enzyme that catalyzes the formation of 1 *μ*mol of glucose per minute under the specified conditions.

Glucose concentration was measured spectrophotometrically with a glucose test based on the Trinder reagent [28]. Glucose is transformed into gluconic acid and hydrogen peroxide by glucose oxidase (GOD). The hydrogen peroxide formed reacts in the presence of peroxidase (POD) with 4-aminoantipyrine and p-hydroxybenzene sulfonate to form a quinoneimine dye, as shown in the following equations.

Hydrolysis of lactose by *β*-galactosidase and glucose determination using a mixture of enzymes, glucose oxidase (GOD), and peroxidase (POD) is as follows:

(2) $$\begin{array}{c} \text{Lactose}\overset{\beta \text{-galactosidase}}{\to }\text{Glucose}+\text{Galactose} \end{array}$$

(3) $$\begin{array}{c} \text{Glucose}+{\text{O}}_{2}+{\text{H}}_{2}\text{O}\overset{\text{GOD}}{\to }\text{Glucotactone}+{\text{H}}_{2}{\text{O}}_{2} \end{array}$$

(4) H2O2+Hydroxybenzoate-Na-4-aminoantipyrine→PODQuinon  complex+H2O

The intensity of the color produced is directly proportional to the glucose concentration in the sample. The assay was performed by mixing 30 *μ*L of a sample of unknown concentration and 3 mL of Trinder reagent, the reaction was left to proceed for 15 min at 37°C, and the absorbance at 510 nm was read. The absorbance was related to the concentration of glucose with a standard calibration curve.

### 3.5. Swelling Studies of Hydrogels

The weight and volume swelling behaviors of dried hydrogels (10 mm diameter, 10 mm thick) were determined by immersion in doubly distilled water or in n-heptane at room temperature. The volume swelling in case of using n-heptane will be the real volume of gel material and not the cylinder volume. The water absorbed was calculated by weighing the samples after wiping, at various time intervals. Swollen gels were weighed by an electronic balance (Sartorius, BP 210S, *d* = 0.1 mg).

Percentage weight swelling (%*q*), the most important parameter in swelling studies, was calculated from the following equation:

(5) $$\begin{array}{c} \%q=\left[\frac{\left(M_{t}-M_{o}\right)}{M_{o}}\right]\times 100, \end{array}$$

where *M*_*t*_ is the mass uptake of the swelling medium at time *t* and *M*_*o*_ is the mass of dry hydrogel.

Percentage volume swelling (%*Q*) was calculated from the following equation:

(6) $$\begin{array}{c} \%Q=\left[\frac{\left(v_{t}-v_{o}\right)}{v_{o}}\right]\times 100, \end{array}$$

where *v*_*t*_ is the volume uptake of the swelling medium at time *t* and *v*_*o*_ is the volume of dry hydrogel. The volume was calculated in terms of weight by using the following equation:

(7) $$\begin{array}{c} V=\frac{\left(W_{a}-W_{h}\right)}{0.684}, \end{array}$$

where *W*_*a*_ is the weight in air and *W*_*h*_ is the weight in n-heptane and the density of n-heptane is equal to 0.684.

For extensive swelling of hydrogels, the following second order kinetics relation was used:

(8) $$\begin{array}{c} \frac{t}{S}=A+Bt. \end{array}$$

The swelling constants were tabulated in Table 1, where *S* is the weight swelling at time (*t*), *B* = 1/*S*_eq_ is the inverse of the maximum or equilibrium weight swelling, *A* = 1/*k*_*s*_ *S*_eq_^2^ is the reciprocal of the initial weight swelling rate (*dS*/*dt*)_*o*_ of the hydrogel, and *k*_*s*_ is the weight swelling rate constant.

The swelling mechanism could be analyzed in swellable polymeric systems by the following equation:

(9) $$\begin{array}{c} \frac{\left(M_{t}-M_{o}\right)}{\left(M_{\text{ eq }}-M_{o}\right)}=Kt^{n}=F, \end{array}$$

where *M*_eq_ is mass of hydrogel at equilibrium. *K* (the swelling constant) and *n* (the swelling exponent) are characteristic parameters of the specific (bioactive agent/dissolution medium) system. By taking natural log of (9) and plotting it against the natural log of time,

(10) $$\begin{array}{c} \ln\frac{\left(M_{t}-M_{o}\right)}{\left(M_{\text{ eq }}-M_{o}\right)}=\ln\;t. \end{array}$$

From (10), the values of *n* and *K* were calculated from the slope and intercept of the plot, where the *n* values determine the mechanism of swelling of each gel formulation.

### 3.6. Morphology of Gel Disks

In this experiment, the surface of the gel disks was examined using scanning electron microscopy (SEM, S-590, HITACHI). Before observation, samples were mounted on metal grids using double-sided adhesive tape and coated by gold under vacuum.

### 3.7. *K*_*m*_ and *V*_max⁡_ of Immobilized Lactase

To obtain the Michaelis-Menten kinetic models adequate for the description of the hydrolysis of lactose by the immobilized enzyme, apparent *K*_*m*_ and *V*_max⁡_ of immobilized *β*-galactosidase were determined for lactose using the Hanes-Woolf plot method:

(11) $$\begin{array}{c} \frac{\left[S\right]}{V_{o}}=\frac{1}{V_{\max}}*\left[S\right]+\frac{K_{m}}{V_{\max}}, \end{array}$$

where [*S*] is the substrate concentration (lactose), *V*_*o*_ is the initial enzyme velocity, *V*_max⁡_ is the maximum enzyme velocity, and *K*_*m*_ is Michaeli's constant and is defined only in experimental terms and equals the value of [*S*] at which *V*_*o*_ equals 1/2*V*_max⁡_. Experimentally, the *K*_*m*_ from the plot is equal to −[*S*], whereas the *V*_max⁡_ is equal to 1/slope. The assay mixture was composed of 5 U of immobilized enzyme and substrate concentration of 20–300 mM at 37°C and pH 4.5 for 3 h.

## 4. Results and Discussion

### 4.1. Covalent Immobilization of Lactase

**Figure 2:**
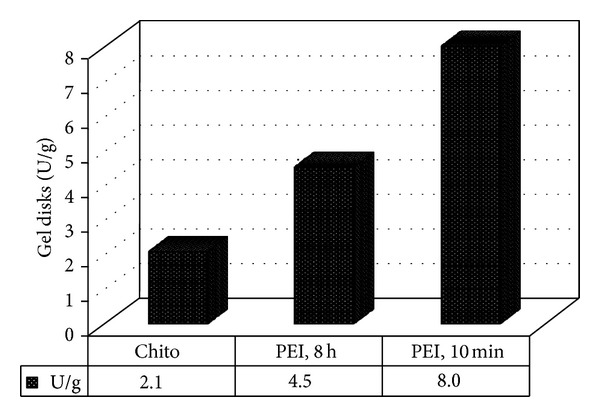
Immobilized lactase onto three gel disks formulations treated as follows: carrageenan/chitosan/glutaraldehyde (Chito), carrageenan/polyethyleneimine, 8 h/glutaraldehyde (PEI, 8 h), and carrageenan/polyethyleneimine, 10 min/glutaraldehyde (PEI, 10 min).

The carrageenan gel was prepared in uniform sheets using the parallel plates apparatus [23] and cut into disks and its surface was modified using partially protonated chitosan or polyethyleneimine. The protonated chitosan or polyethyleneimine amino groups (–NH_3_^+^) formed a polyelectrolyte complex with –OSO_3_^−^ of the carrageenan gel, to incorporate free amino groups as a new functionality [25]. Knowing that the hardening of hydrogels by polyelectrolyte complexation is an interesting alternative to covalently cross-linked hydrogels [28], the free amino groups (–NH_2_) were used to form Schiff's base and covalently immobilize lactase via glutaraldehyde as a mediator. As shown in Figure 2, the gel disks treated with chitosan showed the minimum loading of enzyme, 2.1 U/g, whereas the gel disks treated with PEI for 10 min showed the maximum enzyme loading of 8 U/g. Further soaking of the carrageenan gel disks in PEI till 8 h has an inverse result on the enzyme loading capacity as it decreased from 8 U/g to 4.5 U/g. These results could be regarded as the molecular weight of the polycations to coat and/or penetrate the gel disks. In case of chitosan, it has the highest molecular weight of 150,000 and it was expected to slightly penetrate the gel disks and to remain mostly on the gel surface. Thus, most enzymes were expected to bound to chitosan on the gel surface and on overall it showed the least enzyme loading capacity. With regard to PEI, it has a relatively very small molecular weight of 423, which enables it to easily penetrate the gel disks. Thus, after 10 min, suitable amount of PEI penetrated the gel disks and imparted free amino groups onto the surface of the gel disks as well as inside the gel disks to be used to bind the enzyme via glutaraldehyde as a mediator. Thus, enzymes were immobilized onto and into the gel disks. Further increase of soaking the gel disks in PEI increased the concentration of PEI. It was expected that more enzyme would bind to the gel disks as more free amino groups are present onto and into the gel disks; however, we found that less amount of enzymes were loaded. This could be regarded as the excess PEI onto the gel surface that was used to immobilize the enzyme, which blocked the way of more enzymes to penetrate inside the gel and thus the overall loaded enzymes were less than that after PEI, 10 min.

### 4.2. Hydrogels Swelling

The hydrogel swells when it is brought into contact with water. The water diffuses into the hydrogel and it swells. Diffusion involves migration of water into preexisting or dynamically formed spaces between hydrogel chains. Swelling of the hydrogel involves larger scale segmental motion resulting, ultimately, in an increased distance of separation between hydrogel chains [29].

**Figure 3:**
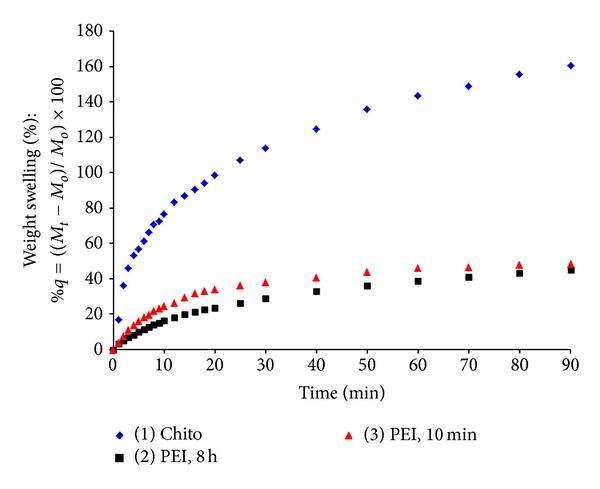
Dynamic weight swelling behavior of hydrogels. Three gel formulations were studied: carrageenan/chitosan/glutaraldehyde (Chito), carrageenan/polyethyleneimine, 8 h/glutaraldehyde (PEI, 8 h), and carrageenan/polyethyleneimine, 10 min/glutaraldehyde (PEI, 10 min).

**Figure 4:**
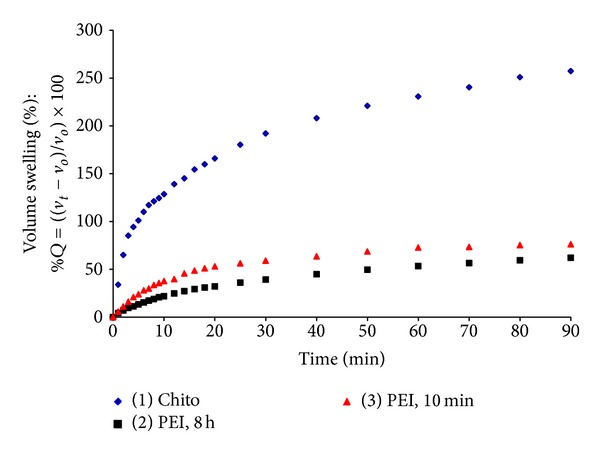
Volume swelling study of hydrogels. Three gel formulations were studied: carrageenan/chitosan/glutaraldehyde (Chito), carrageenan/polyethyleneimine, 8 h/glutaraldehyde (PEI, 8 h), and carrageenan/polyethyleneimine, 10 min/glutaraldehyde (PEI, 10 min).

As shown from Figures 3 and 4, maximum weight and volume swelling percentage were observed for the Chito formulation reaching more than 160 and 260% compared to 45 and 60% for the PEI formulations at 90 min, respectively. The swelling percentage for the PEI formulations at 10 min and 8 h showed no significant difference.

The outstanding increase of the weight swelling percentage for the Chito formulation over the PEIs formulations could be attributed to the high hydrophilic nature of chitosan (free –OH, –NH_2_ groups), which hinders the aggregation of the carrageenan chains and acts to expand the collapsed structure. Thus, chitosan picks up water that diffuses into the polymeric matrix even in the inner regions, increasing the equilibrium water uptake [30]. Correlo et al., 2007, found that the rate and the equilibrium water uptake were proportional to the amount of chitosan in the blend. They regarded that as the loss in the polymer tensile strength as a function of time. In another way, the degradation of properties was directly related to the water uptake of the blends; the higher the water uptake, the higher the degradation. This interpretation goes side by side with our results when we looked at the gel disks' mechanical strength (MS) as shown in Table 1. The MS for the gel disks treated with PEI was 13.3–16.3 kg/cm^2^, whereas that for chitosan was fairly 0.28 kg/cm^2^. These results showed the low mechanical strength of the chitosan formulation and that could be the main reason for its outstanding high rate of swelling in water.

### 4.3. Swelling Kinetics Studies

**Figure 5:**
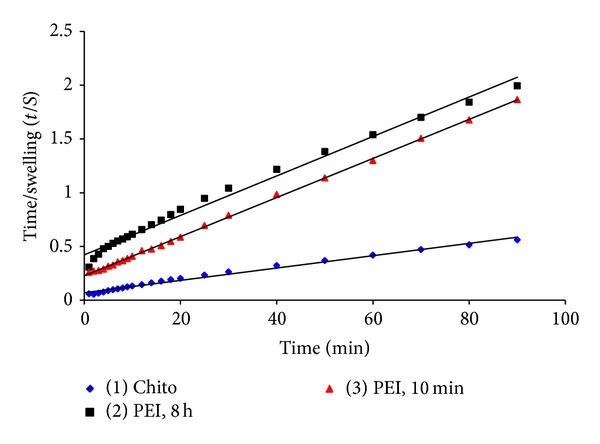
Time/swelling as a function of time for the three gel formulations: carrageenan/chitosan/glutaraldehyde (Chito), carrageenan/polyethyleneimine, 8 h/glutaraldehyde (PEI, 8 h), and carrageenan/polyethyleneimine, 10 min/glutaraldehyde (PEI, 10 min).

The swelling kinetics constants shown in Table 1 have been calculated by plotting *t*/*s* against *t* using the second order kinetics relation (8), *t*/*s* = *A* + *Bt*, as shown in Figure 5.

The regression values for the second order linear plot were calculated from the equation *y* = *Bx* + *A*. According to the data in Table 1, the regression values for the Chito, PEI (8 h), and PEI (10 min) formulations were as follows, respectively:

(12) y=0.0057x+0.0701, where  R2=0.9899,y=0.0183x+0.4224, where  R2=0.9896,y=0.0182x+0.2272, where  R2=0.9995.

It can also be noticed from Table 1 that the values of theoretical equilibrium swelling (*S*_eq_^*a*^) are almost equal to the experimental equilibrium swelling (*S*_eq_^*b*^). However, the swelling equilibrium for the Chito formulation is almost 3-fold that of the PEI formulations. This could be regarded as the high capacity of the Chito formulation to water uptake [30].

The maximum swelling rate constant was clearly observed for the Chito formulation, which reached the value of 439.068 g gel/g water*·*min. This value is far beyond that of the carrageenan gel disks treated with PEI for 10 min and 8 h, which only reached 13.288 and 7.069 g gel/g water*·*min, respectively. These data are in accordance with that observed in Figures 3 and 4 where the Chito formulation has shown the highest weight and volume swelling compared to the PEI formulations.

### 4.4. Swelling Power and Mechanism

A critical analysis of the swelling process reveals that there are two underlying molecular processes: penetration of the solvent molecules into the void spaces in the network and subsequent stretching or relaxation of the network segments. The fundamental equation (*M*_*t*_ − *M*_*o*_)/(*M*_*∞*_ − *M*_*o*_) = *Kt* ^*n*^ defines the following three situations.

1. When the rate of solvent penetration is the slowest and hence is the rate limiting step, the process is called Fickian process, where the value of *n* = 0.5.
2. When the penetration velocity is far greater than the chain relaxation rate, the solvent uptake is proportional to time and this is often called relaxation-controlled case III transport, where the value of *n* = 1.
3. When both the diffusion and polymer relaxation control the overall rate of water uptake, the diffusion mechanism is non-Fickian, where 0.5 < *n* < 1 [31].

**Figure 6:**
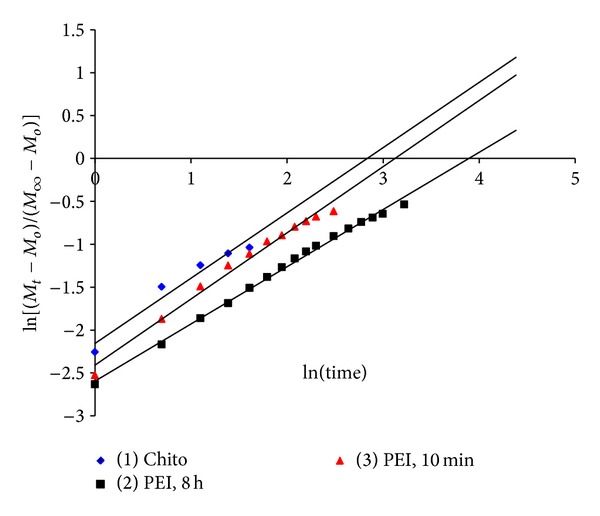
Relation between ln⁡⁡[(*M*_*t*_–*M*_*o*_)/(*M*_*∞*_ − *M*_*o*_)] and time for the three gel formulations: carrageenan/chitosan/glutaraldehyde (Chito), carrageenan/polyethyleneimine, 8 h/glutaraldehyde (PEI, 8 h), and carrageenan/polyethyleneimine, 10 min/glutaraldehyde (PEI, 10 min).

The values of *n* and *K* were calculated from the slope and intercept of the plot in Figure 6 of ln⁡⁡(*M*_*t*_ − *M*_*o*_)/(*M*_*∞*_ − *M*_*o*_) against ln⁡⁡(*t*), respectively. The *n* values for the Chito, PEI 10 min, and PEI 8 h were 0.761, 0.7714, and 0.6667, respectively. By observing these *n* values, where *n* is less than 1 and higher than 0.5, the mechanism of the polymer complexes swelling is probably non-Fickian (anomalous transport). Similar results were obtained by Correlo et al., 2007, when he used blends of chitosan [30]. Correlo et al. found that the water uptake depends on both the diffusion and polymer relaxation, which are controlling the overall rate of water uptake.

### 4.5. Diffusion of Chemicals in the Polymers

Diffusion, *D*, of the chemical in the polymer can be calculated as follows:

(13) $$\begin{array}{c} D=\frac{T}{{\left(t/A\right)}^{1/2}}; \end{array}$$

 
*T* is friction velocity, m/s;
 
*t*_1/2_ is time at which swelling is half the equilibrium swelling, min;
 
*A* is area of the cylinder, cm^2^;
 
*r* is radius of the cylinder, cm.

This equation is based on *T* having a value of 0.049 when half of the equilibrium swelling was achieved as follows:

(14) $$\begin{array}{c} D=\frac{0.049}{{\left(t_{1/2}/4xr2\right)}^{1/2}}. \end{array}$$

By applying (14) to the three gel formulations, we found the diffusion rate to be 39.56, 19.65, and 7.51 (×10^−5^) cm^2^ *·*min^−1^ for the formulations of Chito, PEI 10 min, and PEI 8 h, respectively. Accordingly, we were expecting to see high porosity for the gel formulation of Chito compared to that of the PEI formulations.

### 4.6. Scanning Electron Microscope

**Figure 7:**
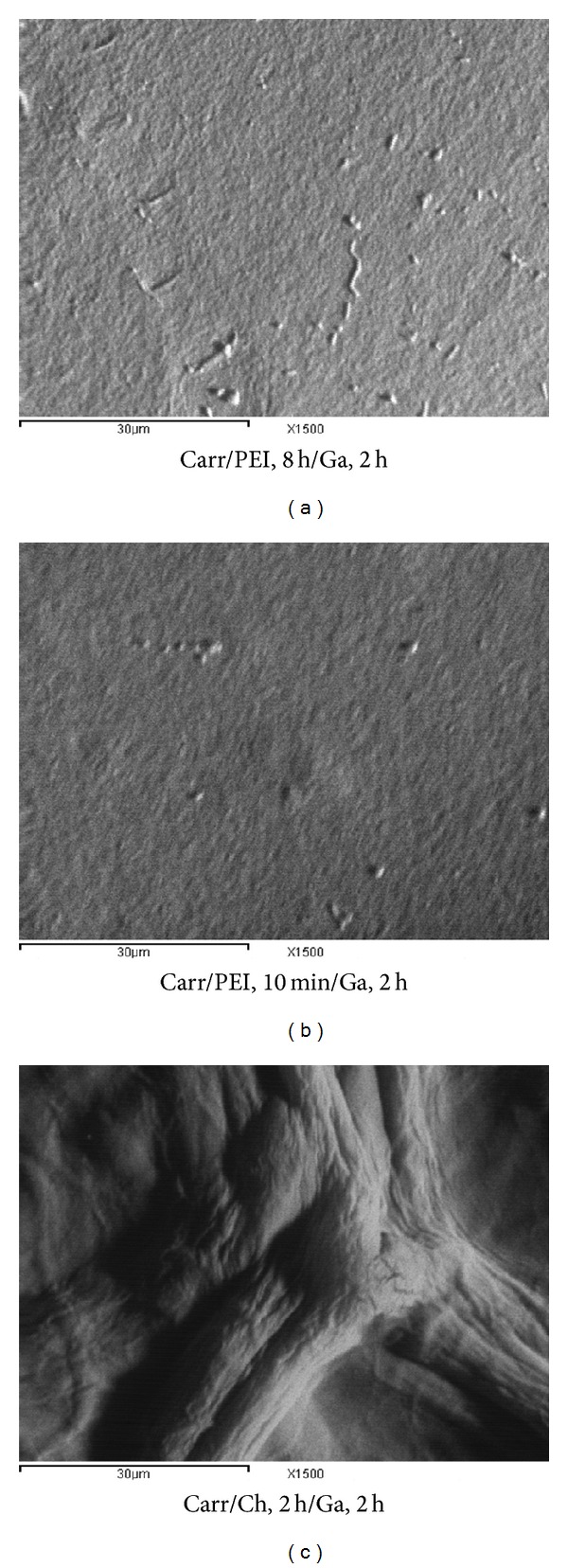
SEM of carrageenan/chitosan/glutaraldehyde, carrageenan/polyethyleneimine, 8 h/glutaraldehyde, and carrageenan/polyethyleneimine, 10 min/glutaraldehyde at ×1500 magnification.

The SEM of the three gel formulations is in accordance with the results obtained from the swelling and the diffusion mechanisms, where the formulation of Chito has shown the maximum swelling and diffusion constant, and these results were assumed to be related to the gel surface. As shown in Figure 7, as expected, the formulation of Chito has the highest and biggest cavities and grooves as well as the largest surface area compared to the PEI formulations.

### 4.7. Kinetic Constants

**Figure 8:**
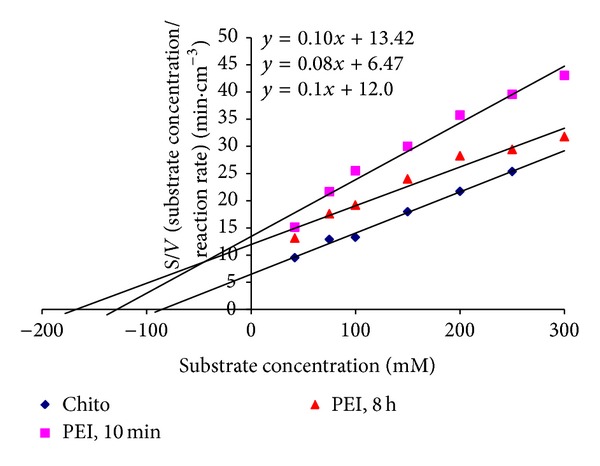
*K*_*m*_ and *V*_max⁡_ for the three gel formulations: carrageenan/chitosan/glutaraldehyde (Chito), carrageenan/polyethyleneimine, 8 h/glutaraldehyde (PEI, 8 h), and carrageenan/polyethyleneimine, 10 min/glutaraldehyde (PEI, 10 min).

Hanes-Woolf plot method was used to calculate the kinetic constants of immobilized lactase as shown in Figure 8. The calculated values for the three gel formulations, Chito, PEI 10 min, and PEI 8 h, were tabulated in Table 2. The Chito formulation has shown the smallest *K*_*m*_ value of 85.51 mM compared to 134.88 and 177 mM for the PEI 10 min and PEI 8 h, respectively. On the other hand, there were no many differences between the *V*_max⁡_ values for the three gel formulations, where their values ranged between 10 and 14.8 min*·*cm^−3^.

### 4.8. Correlation between the *K*_*m*_ of the Enzyme and the Gel Swelling Kinetics and SEM

In this study, we prepared three gel formulations based on treated carrageenan gel disks with chitosan or PEI followed by glutaraldehyde to covalently immobilize lactase. The data in Table 3 could summarize the conclusion of this paper.

As shown in Table 3, the two formulations of Chito and PEI, 8 h, represent the results with the maximum (italic font) and minimum (bold font) values per column. For example, the Chito formulation represents the lowest value of Michaeli's constant (*K*_*m*_) of 85.51 mM and the maximum values for the swelling rate constant (439.068 ggel/gwater*·*min), the swelling power (160.3%), and the diffusion rate (39.56 × 10^−5^ cm^2^/min), whereas the formulation of PEI, 8 h, has shown completely the opposite trend, where it represents the highest value for Michaeli's constant (*K*_*m*_) of 177 mM and the minimum values for the swelling rate (7.069 ggel/gwater*·*min), the swelling power (45.1%), and the diffusion rate (7.51 × 10^−5^ cm^2^/min). The SEM is also supporting this trend as it showed the maximum surface area for the Chito formulation and the minimum one for the PEI 8 h formulation. From these values, we could assume the following correlations: [Surface area] *α* [Swelling rate (*K*_*s*_)] *α* [Swelling power (*q*%)] *α* [Gel surface area] *α* [Diffusion rate] 1/*α* [Michaeli's constant (*K*_*m*_)].

Although this correlation is based on polymers with different surface topologies and does not show different pore sizes, it goes well with the finding of Koilpillai et al., 1990 [32], who observed that the surface microenvironment of the carriers is also influenced by the porosity. For example, increasing the porosity and the specific surface of a fibre-based biocatalyst reduced *K*_*m*_ and the energy of activation for hydrolysis of benzyl penicillin increased *V*_max⁡_ [33], indicating that porosity is closely related to diffusion constraints. However, in our case the *V*_max⁡_ was almost the same for the gel formulations.

## 5. Conclusions

In this study we prepared three gel disk formulations based on Chito and PEI. The PEI has shown a better enzyme loading capacity over the Chito formulation. However, the PEI formulations have shown the highest *K*_*m*_ value. By studying the swelling kinetics of the three formulations we found that the Chito formulation has shown a far beyond swelling rate constant of 62 times that of PEI 8 h and of 33 times that of PEI 10 min gel disk formulations. These results were highly supported by the SEM of the three formulations, which showed an outstanding increase for the surface area of the Chito formulation over the PEI formulations. Furthermore the diffusion rate for the Chito formulation was 2–5 times that of the PEI formulations, which assumes the minimum of substrate/product diffusion limitation. By comparing our finding with other authors, the *K*_*m*_ values of the immobilized enzyme could be controlled by increasing either the carrier's surface area or/and the pore size. However, the *V*_max⁡_ can only increase when the pore sizes increase. These results are highly encouraged to be further explored in biomedical applications such as tissue engineering and drug delivery systems.

## Figures and Tables

**Table 1:** Swelling constants *A* and *B*, theoretical swelling equilibrium *S*_eq_^*a*^, experimental swelling equilibrium *S*_eq_^*b*^, initial swelling rate, swelling rate constant *K*_*s*_, weight swelling power %*q*, volume swelling power %*Q*, and gel disks mechanical strength.

Formulation	*A*	*B*	*S*_eq_^*a*^ Theoretical swelling equilibrium (g_water_/g_gel_) (1/*B*)	*S*_eq_^*b*^ Experimental swelling equilibrium (g_water_/g_gel_)	Initial swelling rate (g_gel_/g_water_ *·*min) (1/*A*)	*K*_*s*_ *·* 10^3^ Swelling rate constant (g_gel_/g_water_ *·* min)	%*q* Weight swelling power	%*Q* Volume swelling power	MS (kg/cm^2^)
Chito	0.0701	0.0057	**175.4**	**180.5**	**14.27**	**439.068**	**160.3**	**257.2**	**0.28**
PEI, 8 h	0.4224	0.0183	**54.6**	**53.3**	**2.37**	**7.069**	**45.1**	**62.1**	**16.3**

PEI, 10 min	0.2272	0.0182	54.9	51.3	4.40	13.288	48.3	76.2	**13.35**

**Table 2:** Kinetic constants of immobilized lactase onto the three gel formulations.

Formulation	*K*_*m*_ (mM)	*V*_max⁡_ (min*·*cm^−3^)
Chito	**85.51**	13.2
PEI, 8 h	**177**	**14.8**

PEI, 10 min	134.88	10

**Table 3:** Correlation between the enzyme kinetic constant (*K*_*m*_) and the swelling and diffusion behavior of the gel formulations.

Formulation	*K*_*m*_ (mM)	*K*_*s*_ *·* 10^3^ Swelling rate constant (g_gel_/g_water_ *·*min)	Diffusion *D* ∗ 10^5^ cm^2^/min	Swelling power *q*%
Chito	**85.51**	*439.068 *	*39.56 *	*160.3 *
PEI, 8 h	*177 *	**7.069**	**7.51**	**45.1**

PEI, 10 min	134.88	13.288	19.56	48.3
